# Economic Synergistic Development of Guangdong-Hong Kong-Macao Greater Bay Area Urban Agglomeration: Based on Composite System

**DOI:** 10.1155/2022/7677188

**Published:** 2022-09-13

**Authors:** Juan Yang

**Affiliations:** ^1^Research Institute of Innovation Competitiveness of Guangdong, Hong Kong and Macao Bay Area, Guangdong University of Finance & Economics,Guangdong, Guangzhou 510320, China; ^2^Foshan Modern Service Industry Research Institute, Guangdong University of Finance & Economics,Guangdong, Guangzhou 510320, China

## Abstract

Synergistic development is the only way which must be passed and a key point to achieve high-quality economic development. This paper regards regional synergetic development as a composite system, builds up the evaluation indicator system, and calculates the level of economic synergetic development of Guangdong-Hong Kong-Macao Greater Bay Area Urban Agglomeration, by using the collaborative degree model of composite system. The results show that each subsystem of the composite system has a high degree of order from 2007 to 2019, but compared with Beijing-Tianjin-Hebei urban agglomeration and Yangtze River Delta urban agglomeration, the level of economic collaborative development of Guangdong-Hong Kong-Macao Greater Bay Area urban agglomeration in 2008–2019 is relatively low and has large spatial differences. The main reason is that under the background of “one country, two systems” policy, the institutional differences between Guangdong, Hong Kong, and Macao have not been effectively linked up and synergetic, Greater Bay Area urban agglomeration has not yet formed an organic whole, and the synergy effect of mutual support and promotion is relatively weak. Based on this, we should seize the great historical opportunity of the construction of Guangdong-Hong Kong-Macao Greater Bay Area, accelerate the construction of the mechanism for the synergetic economic development of the three areas, accelerate the establishment of an integrated market, build a reasonable division of labor system and collaborative innovation system, and jointly promote the synergetic economic development of Greater Bay Area.

## 1. Introduction

Over the past 40 years of reform and opening up, the cooperation between Guangdong, Hong Kong, and Macao has been gradually deepened, which has led to a notable success in the economic and social development of Guangdong, and also accelerated the transformation of Hong Kong and Macao into a service-oriented economy. After maintaining a sound and stable growth trend for many years, the economies of the Pearl River Delta, Hong Kong, and Macao have encountered new bottlenecks in structural adjustment and development power. It is required to start from a more systematic and holistic development approach to expand space and inject new momentum into the economic development of the three places. On February 18, 2019, the *Outline Development Plan for the Guangdong-Hong Kong-Macao Greater Bay Area* was released, marking that this national strategy, which was personally planned, deployed, and promoted by General Secretary Xi Jinping, has entered the stage of full implementation. In the *Outline*, “promoting the synergetic development of regional economy” and “exploring a new model of synergetic development” are clearly defined as the guiding ideology and strategic positioning. The synergetic development here is a way to fully explore the potential advantages of various regions through efficient and orderly integration, promote the orderly flow and optimal allocation of factor resources to the greatest extent in space, and promote the formation of more effective and rational division of labor and cooperation, so as to release the development mode of the growth potential of new economy [1]. Therefore, in order to win competitive advantages through the allocation of resources in a wider space, effectively break the bottleneck of economic development in the three places, and accelerate the release of economic growth potential, the synergetic economic development of Guangdong-Hong Kong-Macao Greater Bay Area urban agglomeration is particularly important.

## 2. Literature Review

The concept of synergy was first put forward by German scholar Harmann Haken [2]. In the synergetics theory founded by him, it is mentioned that the structure, behavior, and characteristics of any system are not the simple or direct summation of the subsystems within the system. There is a nonlinear relationship of mutual influence, mutual restriction, and mutual cooperation among subsystems, which will present a certain degree of regular “synergy” effect, so that the subsystems will change from disorder to order, and low-level to high-level, and reach the effect of 1 + 1 > 2. When “synergy” is applied to regional economics, it refers to the development trend of “mutual beneficial symbiosis and win-win cooperation” between various parts in the region [3].

With the proposal of China's regional development strategy, the synergetic development of major urban agglomerations has attracted the attention of scholars. They believe that the synergetic development of the Beijing-Tianjin-Hebei urban agglomeration mainly faces problems such as the large economic development gap between the three places, unclear functional positioning, unreasonable industrial division of labor, lagging infrastructure, lack of collaborative governance mechanism, environmental degradation, and so on. The reasons for these problems mainly come from the internal urban differences, fiscal and tax separation system, the inequality of administrative division, and level [4]. At present, the Yangtze River Delta urban agglomeration has formed a multi-core circle structure with Shanghai as the core and Nanjing, Hangzhou, and Suzhou as the sub-cores, which will boost the synergetic development of the region. However, the problems in the aspects such as the division of labor, environment, transportation, and other fields among cities need to be broken through from the three levels of government, market, and society [5]. In terms of the empirical aspect, Xiao [6], Qiu, and Luo [7] measured the level of synergetic development in different regions by constructing an indicator system with the application of the analytic hierarchy process, principal component analysis, composite system model, etc. In recent years, Zhou [8] and others proposed that the similarity of resource endowments in the Pearl River Delta made the convergence of industrial structure prominent in the synergetic development of Guangdong, Hong Kong, and Macao. The research group of Guangdong-Hong Kong-Macao Greater Bay Area Research Institute of Guangdong University of Foreign Studies believes that the key to the construction of Guangdong-Hong Kong-Macao Great Bay Area is “synergetic development,” and the biggest problem is the construction of multiple synergetic mechanisms under the background of “one country, two systems, three customs areas, three jurisdictions, and multiple centers”[9]. In addition, the restricted factor flow, weak synergetic innovation, and overcapacity are also the reasons that affect the synergetic economic development of Greater Bay Area [10]. Chen, Lin [11], Xiang, and Yang [12] conducted an empirical study on the industrial synergetic development of Guangdong-Hong Kong-Macao Great Bay Area by using the grey correlation analysis method, which further confirmed the convergence of the industrial structure of the Great Bay Area.

The above content has laid a good foundation for the work of the paper, but it mainly focuses on the problems existing in the economic synergetic development of urban agglomeration in the Greater Bay Area. Moreover, the research on the current situation of its synergetic development is relatively superficial and general, lacking data support and quantitative analysis.

The paper attempts to regard the economic synergetic development of Guangdong-Hong Kong-Macao Greater Bay Area urban agglomeration as a composite system, builds a regional economic synergetic development indicator system, quantifies the economic synergetic development level of Guangdong-Hong Kong-Macao Greater Bay Area urban agglomeration from 2008 to 2019 with the help of the synergy degree model of the composite system, and accurately and objectively reflects the current situation of economic synergetic development of Greater Bay Area urban agglomeration through the comparison with other major urban agglomerations in China. Combined with the institutional background of “one country, two systems” po, the paper analyzes and discusses the root causes of the current situation, and then puts forward corresponding policy recommendations. This has certain theoretical and practical significance for the practice and innovation of the “one country, two systems” theory, the high-quality economic development of Guangdong-Hong Kong-Macao Great Bay Area, and the construction of world-class bay area and urban agglomeration.

## 3. Construction of Indicator System and Model Selection

In this paper, the synergetic development of regional economy is regarded as a composite system, and the level of synergetic development of regional economy is calculated by constructing an evaluation indicator system and using the synergy degree model.

### 3.1. Construction of Indicator System

Considering the differences of the social and economic statistical methods and indicator system between Hong Kong and Macao Special Administrative Regions and the mainland, in order to ensure the availability and consistency of the data, the paper constructs an evaluation indicator system of regional economic synergetic development, including the following five subsystems, a total of 23 indicators.

#### 3.1.1. Economic Scale Subsystem

Regional economic development is first manifested in the expansion of economic scale, namely, the improvement of the production capacity of the region, the expansion of market scale, the improvement of the ability of government to provide public goods and services, and the increase of social consumption, and production input. Therefore, the regional GDP, fiscal revenue, total retail sales of consumer goods, fixed asset investment, and total employment are selected to manifest the economic scale.

#### 3.1.2. Economic Quality Subsystem

Another manifestation of regional economic development is the improvement of development quality, including the improvement of regional economic growth potential, the optimization of industrial structure, and the improvement of production efficiency, living standards, and employment levels. Therefore, per capita GDP, the proportion of the tertiary industry in GDP, labor productivity, final consumption rate, and unemployment rate are selected as the indicators of economic quality subsystem.

#### 3.1.3. Sustainable Development Subsystem

Except for the expansion of “quantity” and the improvement of “quality,” regional economic development also manifests the sustainability of growth mode. The sustainable development subsystem is manifested by the proportion of scientific research expenditure, education expenditure, environmental protection expenditure in GDP, and the number of sickbeds per 10,000 people.

#### 3.1.4. Element Flow Subsystem

Free and convenient flow of elements, and rational and effective allocation of resources are one of the important prerequisites for realizing regional complementary advantages and economic development. The average daily passenger flow, average daily freight volume, per capita FDI, and the number of mobile phones per 100 people are selected as the representatives of the element flow of personnel, goods, funds, and information, respectively.

#### 3.1.5. Market Environment Subsystem

A free and open market environment with fair competition is the foundation of regional economic development. The paper intends to consider the market environment subsystem from five aspects: opening up of foreign trade, opening up of foreign capital, opening up of tourism, government scale, and financial environment, which are, respectively, divided into ratio of dependence on foreign trade, opening ratio of foreign capital, opening ratio of tourism, proportion of government consumption expenditure in GDP, and per capita loan scale, respectively.

The above indicators are listed in Table 1.

### 3.2. Model Selection

The synergy degree model of composite systems is based on the order parameter and slaving principle, and effectively quantifies the process of complex systems from disorder to order by using the order degree of each subsystem and the synergy degree model of composite system [13].

#### 3.2.1. Model of Ordering Degree of Subsystem

A composite system *A*=(*A*_1_, *A*_2_, ⋯⋯, *A*_*n*_) of synergetic development of regional economy is established. It supposes that the order parameter corresponding to the subsystem *A*_*i*_ is *b*_*i*_=(*b*_*i*1_, *b*_*i*2_, ⋯, *b*_*in*_). When the value of order parameter *b*_*i*_ is positive, the larger the value of its order parameter component *b*_*i*1_,*b*_*i*2_,…,*b*_*ik*_, the higher the degree of order is; on the contrary, the degree of order will be lower. The order degree of order parameter components can be obtained by the following model:

(1) $$\begin{array}{c} \mu \left(b_{ij}\right)=\left\{\begin{array}{cc} \frac{b_{ij}-\alpha_{ij}}{\beta_{ij}-\alpha_{ij}}\text{,} & \;j\in \left[1,k\right]\text{,}\\ \frac{\beta_{ij}-b_{ij}}{\beta_{ij}-\alpha_{ij}}\text{,} & j\in \left[k+1,n\right]\text{.} \end{array}\right. \end{array}$$

Among them, *μ*(*b*_*ij*_) ∈ [0,1], the greater the value is, the greater the contribution of the subsystem to the composite system is. *∂*_*ij*_ and *β*_*ij*_ are the critical upper limit value and lower limit value of the order parameter, respectively. In the paper, the minimum and maximum values of the order parameter in the research period are selected as representatives. From the perspective of the whole composite system, the contribution of each order parameter component to the subsystem is closely related to its combination form and weight, namely, it needs to be realized through their own integration of *μ*(*b*_*ij*_). The paper adopts the linear weighting method to calculate the order degree of subsystem.

(2) $$\begin{array}{c} \mu \left(b_{i}\right)=\overset{n}{\underset{j=1}{\sum }}\omega_{j}\mu \left(b_{ij}\right)\omega_{j}\geq 0,\\ \overset{n}{\underset{j=1}{\sum }}\omega_{j}=1, \end{array}$$

*μ*(*b*_*i*_) is the order degree of the subsystem, *μ*(*b*_*i*_) ∈ [0,1]. The larger *μ*(*b*_*i*_) is, the higher the order degree of the subsystem is, and vice versa. *ω*_*j*_is the indicator weight which is determined by entropy method. First, assuming that there are *m* cities and *n* evaluation indicators, the order degree probability distribution of order parameter component is

(3) $$\begin{array}{c} P_{ij}=\frac{\mu \left(b_{ij}\right)}{\sum_{i=1}^{m}\mu \left(b_{ij}\right)}\text{.} \end{array}$$

As mentioned in equation (3), *μ*(*b*_*ij*_) ∈ [0,1], the entropy value of the *j*_th_ indicator of the subsystem *A*_*i*_ is

(4) $$\begin{array}{c} d_{j}=-k\overset{m}{\underset{i=1}{\sum }}P_{ij}\ln P_{ij}. \end{array}$$

Among them, *k*=1/ln *m*, then, the entropy weight *ω*_*j*_ of the *j*_th_ indicator can be defined as

(5) $$\begin{array}{c} \omega_{j}=\frac{1-d_{j}}{\overset{m}{\underset{i=1}{\sum }}\left(1-d_{j}\right)}\text{.} \end{array}$$

#### 3.2.2. Synergy Model of the Composite System

Assuming that the order degree of the subsystem *A*_*i*_ of the composite system of regional economic synergetic development in the period *t*_0_ is *μ*^0^(*b*_*i*_), and the order degree in the period *t*_*i*_ is *μ*^1^(*b*_*i*_), then the synergy degree *E* of the regional economic synergetic development system is

(6) $$\begin{array}{c} E=u\sqrt[{n}]{\prod_{i=0}^{n}\left[|\mu^{1}\left(b_{i}\right)|-|\mu^{0}\left(b_{i}\right)|\right]}\text{.} \end{array}$$

At the same time， $u=\left\{\begin{array}{c} 1，\mu^{1}\left(b_{i}\right)\geq \mu^{0}\left(b_{i}\right)\\ -1,\mu^{1}\left(b_{i}\right)<\mu^{0}\left(b_{i}\right) \end{array}\right.\text{.}$

The value of *E* is [-1, 1]. The higher the value is, the higher the level of regional economic synergetic development is, and vice versa. Parameter *u* measure the synergy direction of each subsystem. When the value of *u* is 1, the synergy degree *E* is positive, which indicates that the order degree of each subsystem is in the rising stage, and the composite system is in the synergy and order stage; When the value of *u* is -1, *E* is negative, indicating that the order degree of at least one subsystem declines, and the composite system is in the unstable or unsynergetic stage.

## 4. Calculation Results and Analysis

### 4.1. Data Sources and Description

The paper takes the panel data from 2007 to 2019 of the 11 cities of Guangdong-Hong Kong-Macao Greater Bay Area urban agglomeration, namely Guangzhou, Shenzhen, Zhuhai, Foshan, Zhongshan, Zhaoqing, Dongguan, Huizhou, and Jiangmen, as well as Hong Kong and Macao as the main research samples. The data sources of each indicator include *Guangdong Statistical Yearbook*, *China Urban Statistical Yearbook*, *Hong Kong Statistical Yearbook*, and *Macao Statistical Yearbook* over the years.

Considering that the currency units used by cities in the Pearl River Delta, Hong Kong, and Macao are not the same, in order to facilitate the unification and calculation of indicators, the exchange rates of each year are converted into RMB for indicator statistics and analysis.

For the missing original data, the mean method is adopted for processing. The indicator data that are not directly published in the statistical yearbook or annuals is obtained by calculation.

### 4.2. Calculation Results

#### 4.2.1. Changes of Order Degree of Subsystems in the Synergistic Economic Development of Guangdong-Hong Kong-Macao Greater Bay Area Urban Agglomeration

According to the calculations of formulas (1)to (5), the order degree of each subsystem is obtained. As shown in Table 2, the order degree of each subsystem of the economic synergetic development composite system of Guangdong-Hong Kong-Macao Greater Bay Area urban agglomeration manifested a rising trend in the years under investigation. Among them, the economic scale subsystem has the highest order degree and the fastest increase, steadily increasing from 0.4818 in 2007 to 0.8157 in 2019. The order degrees of economic quality subsystem and element flow subsystem increased from 0.4262 and 0.4000 to 0.6794 and 0.6395, ranking second and third, respectively. Then, it is the market environment subsystem, which increased from 0.3592 in 2007 to 0.5350 in 2019. In contrast, the sustainable development subsystem has the lowest order degree and the most obvious fluctuation. That is, it increased from 0.2737 in 2007 to 0.5102 in 2013, then decreased to 0.3807 in 2016, and finally increased to 0.4161 in 2019. On the whole, the subsystems of the economic synergetic development composite system of Guangdong-Hong Kong-Macao Greater Bay Area have a relatively high order degree.

#### 4.2.2. Temporal Changes in the Level of Synergistic Economic Development of Guangdong-Hong Kong-Macao Greater Bay Area Urban Agglomeration

Based on Table 2, the synergy degree of the composite system of economic synergetic development of Guangdong-Hong Kong-Macao Greater Bay Area from 2008 to 2019 is calculated through formula (6), which is the level of synergetic development (see Table 3). The results show that the level of synergetic economic development of Guangdong-Hong Kong-Macao Greater Bay Area urban agglomeration increased from −0.0123 in 2008 to 0.0786 in 2011, then decreased to −0.0875 in 2012, then continued to increase, and finally reached the maximum value of 0.216 in 2019. According to the level and classification criteria of regional economic synergetic development in Table 4, the level of economic synergetic development of Greater Bay Area urban agglomeration was in the non-synergetic stage from 2008 to 2009, entered the low-level synergetic stage from 2010 to 2011, fell to the non-synergetic stage again from 2012 to 2013, and entered the primary synergetic stage from the low-level synergetic stage from 2014 to 2019. It can be seen that from 2008 to 2019, the synergetic economic development level of Guangdong Hong Kong Macao Greater Bay Area Urban Agglomeration manifested a trend of gradual improvement, and the change was more obvious, namely, from the non-synergetic stage to the primary synergetic stage.

#### 4.2.3. Spatial Changes in the Level of Synergistic Economic Development of Guangdong-Hong Kong-Macao Greater Bay Area Urban Agglomeration

Referring to the method of Guo Shipping [14], the paper divides the Greater Bay Area urban agglomeration into three groups, namely, core cities, sub-core cities, and non-core cities. Among them, Guangzhou, Shenzhen, and Hong Kong are the core cities, Foshan, Zhuhai, Dongguan, and Zhongshan are the sub-core cities, and Huizhou, Zhaoqing, Jiangmen, and Macao are the non-core cities. Then, the level of economic synergetic development of each group of cities is calculated separately to explore the temporal and spatial changes of economic synergetic development of Greater Bay Area urban agglomeration. The specific method is the same as above. As shown in Table 3, the level of synergetic economic development of cities in each group is on the rise, but there is a certain gap between them. Among them, the sub-core cities have the highest level of synergetic economic development, with core cities ranking second and non-core cities ranking third. Specifically, the level of synergetic economic development of sub-core cities in 2008 was 0.2555, and then rose to 0.3387 in 2010. After a slight adjustment, it steadily increased to 0.4024 in 2019, realizing the transition from the primary synergetic stage to the intermediate synergetic stage; the change trend of the economic synergetic development level of core cities was roughly the same as that of sub-core cities, rising from 0.0838 in 2008 to 0.2534 in 2019, from low-level synergy to primary synergy; non-core cities increased from −0.0774 in 2008 to 0.1143 in 2019, from the unsynergetic stage to the reluctantly synergetic stage.

#### 4.2.4. Changes in the Level of Synergistic Economic Development of Different Urban Agglomerations

In order to further objectively and accurately grasp the economic synergetic development of Guangdong-Hong Kong-Macao Greater Bay Area urban agglomeration, the paper also measures the economic synergetic development level of Beijing-Tianjin-Hebei urban agglomeration and Yangtze River Delta urban agglomeration, using the same method as before. Beijing-Tianjin -Hebei urban agglomeration and Yangtze River Delta urban agglomeration are two regions except Guangdong Hong Kong Macao Great Bay Area in China that are expected to become world-class urban agglomerations, with relatively large economic development scale, relatively high speed, and relatively high population density. The statistical data of these cities are mainly from the *China Statistical Yearbook* and *China Urban Statistical Yearbook* over the years, as well as the statistical yearbooks of provinces and cities. As shown in Table 5, the synergetic economic development trends of the three urban agglomerations were all from low to high from 2008 to 2019, but there was a large gap in the level of synergetic development. In 2008, the levels of synergetic economic development of urban agglomerations in the Yangtze River Delta, Beijing-Tianjin-Hebei, and Guangdong-Hong Kong-Macao Greater Bay Area were 0.2493, 0.1167, and -0.0123, respectively, which were in the stage of primary synergy, reluctant synergy, and non-synergy, respectively; in 2014, they increased to 0.4375, 0.2593, and 0.0843, respectively, and entered the stage of intermediate synergy, primary synergy, and low-level synergy in turn; in 2019, the economic synergetic development levels of the Yangtze River Delta urban agglomeration and the Beijing-Tianjin-Hebei urban agglomeration increased to 0.5194 and 0.4003, respectively, both in the intermediate synergetic stage; the synergetic economic development level of Guangdong-Hong Kong-Macao Greater Bay Area urban agglomeration is 0.2160, entering the primary collaborative stage. It can be seen that among the three urban agglomerations, the Yangtze River Delta urban agglomeration has the highest level of economic synergetic development, followed by Beijing-Tianjin-Hebei region, and the Guangdong-Hong Kong-Macao Greater Bay Area urban agglomeration has always been at the lowest level.

### 4.3. Result Analysis

From the perspective of the principle of synergetics, only when there is a close relationship between subsystems that promote and depend on each other can the composite system produce a synergistic effect in which the overall function is greater than the simple summation of local functions. Although the subsystems of the composite system have a relatively high order degree, the overall level of the synergetic economic development of Guangdong-Hong Kong-Macao Greater Bay Area is at a low level, lagging behind the Beijing-Tianjin-Hebei urban agglomeration and the Yangtze River Delta urban agglomeration, and there are large spatial differences. This shows that the degree of synergy between the urban agglomerations in Greater Bay Area is low, and the relationship of “mutually beneficial symbiosis and win-win cooperation” has not yet been formed. The reason for this situation mainly comes from the institutional differences between Guangdong, Hong Kong and Macao under the background of “one country, two systems” policy [15].

Different from other urban agglomerations, Guangdong-Hong Kong-Macao Greater Bay Area is a cross-border cooperation of three independent tariff zones within the same sovereign country under the background of “one country, two systems” policy, and implements different political and social systems. This institutional framework ensures that the Pearl River Delta, Hong Kong, and Macao share the same fundamental and long-term interests, and lays a solid foundation for the synergetic development of the economies of the three places, but many institutional differences have also been produced, bringing many imbalances and asymmetries to the economic cooperation among the three regions. For example, Hong Kong and Macao are free ports with a high degree of internationalization and rule of law, perfect market economy rules, and little government intervention in the economy. However, the Pearl River Delta has not been formally integrated with the world economic system, the level of opening to the outside world and the development of market economy need to be improved, and the government-led characteristics are still obvious. At the same time, Guangdong-Hong Kong-Macao Greater Bay Area belongs to three independent tariff areas, so the circulation of goods in the Greater Bay Area faces different trade policies, tax arrangements, and regulatory systems; people from the three places need to apply for visas, so the number of visits is limited, and there are also restrictions on academic qualifications, quotas, duration of stay, social welfare, professional qualification recognition, and so on; the financial supervision systems of Guangdong, Hong Kong, and Macao are independent, the financial markets are isolated from each other, and the three currencies are not circulating with each other; the mainland has opened up to Hong Kong and Macao in the fields of telecommunications and Internet with conditions. The residents of Hong Kong and Macao need to switch networks and pay roaming charges to and from the Pearl River Delta. In order to adapt to or overcome these institutional differences, the market entities of Guangdong, Hong Kong, and Macao need to pay additional institutional transaction costs, resulting in that business activities cannot be carried out on the principle of profit maximization, which reduces the willingness to cooperate across regions. These institutional transaction costs may also cause market segmentation and monopoly, breed local protectionism and departmentalism, form intra-regional trade barriers, and reduce the efficiency and quality of resource allocation in the entire Great Bay Area, which is not conducive to the formation of an integrated market.

Since the return of Hong Kong and Macao to China, in order to solve the problem of inclusiveness under the “one country, two systems” policy, a multi-level institutional arrangement from the central government to local levels has been carried out for many years, which helps to promote the in-depth cooperation among Guangdong, Hong Kong, and Macao, but the connection and synergy of the systems among the three places have never achieved a major breakthrough. Driven by a series of institutional arrangements including CEPA and Guangdong Pilot Free Trade Zone, the division of labor and cooperation in the service industry among Guangdong, Hong Kong, and Macao has achieved certain results. However, as the market opening and access conditions, and business environment of the three places have not been effectively connected, the division of labor and cooperation in the service industry among Hong Kong, Macao, and the Pearl River Delta still remains in the traditional fields such as transportation service, tourism service, and business service. Hong Kong's advantageous fields such as insurance, law, accounting, and construction have not really achieved effective connection and complementary advantages with the manufacturing industry in the Pearl River Delta. In addition, with the convergence of the economic development structure of the Pearl River Delta, Hong Kong, and Macao, as well as the improvement of the mainland's infrastructure, the three places have launched homogeneous competition in the fields of finance, logistics, exhibition, and scientific and technological innovation. Such a division of labor has not only prevented Guangdong, Hong Kong, and Macao from becoming an economic community and interest community with shared risks and interests but also brought too much internal friction to the construction of the Great Bay Area, which reduces the overall effect of economic development.

## 5. Conclusions and Policy Recommendations

The paper constructs an evaluation indicator system of regional economic synergetic development, applies the synergy degree model of the composite system to calculate the economic synergy level of Guangdong-Hong Kong Macao Greater Bay Area urban agglomeration from 2008 to 2019, and compares it with Beijing-Tianjin-Hebei urban agglomeration and Yangtze River Delta urban agglomeration. The following conclusions are drawn: (1) The economic synergy development level of Guangdong-Hong Kong-Macao Greater Bay Area has changed significantly in different periods, showing a gradual upward trend on the whole from non-synergy stage to primary synergy stage; (2) There are large spatial differences in the level of synergetic economic development of Guangdong-Hong Kong-Macao Greater Bay Area urban agglomeration. The level of synergetic economic development of sub-core cities is the highest, followed by core cities and non-core cities; (3) Through the comparison, the synergetic economic development of Guangdong-Hong Kong-Macao Greater Bay Area urban agglomeration has the roughly same trend as the Yangtze River Delta urban agglomeration and Beijing Tianjin Hebei urban agglomeration, but it is still at a low level. The main reason for these current situations is that the institutional differences under the background of “one country, two systems” policy in the urban agglomeration of Guangdong-Hong Kong-Macao Greater Bay Area have not been effectively connected and synergetic, resulting in loose cooperation among the three places, and the synergistic effect of mutual and joint cooperation is still relatively weak. Based on this, the paper puts forward the following countermeasures:

1. Constructing a mechanism for synergetic economic development and uniting the awareness of common development of all parties: giving full play to the advantages of “one country, two systems” policy, constructing a common framework across the institutional gap, planning and laying out the development of Greater Bay Area as a whole, and promoting the three regions from complementary advantages to integration of advantages are important guarantees and top priority for the synergetic economic development of Greater Bay Area urban agglomeration. It is suggested to establish a “Leading Group for the Synergistic Economic Development of Guangdong-Hong Kong-Macao Greater Bay Area Urban Agglomeration” as the organization for overall planning, decision-making and synergy, which is responsible for promoting the synergy among the cities in the Greater Bay Area at the national level; the “Synergy and Management Organization for the Synergistic Economic Development of Guangdong-Hong Kong-Macao Greater Bay Area Urban Agglomeration” should be established as the daily leadership and executive organization for the synergetic economic development of Greater Bay area to be responsible for the implementation and implementation of various plans and designs, the formulation of supporting supplementary agreements and implementation rules, and the guidance of specific work; the “Supervision and Management Organization for the Synergistic Economic Development of Guangdong-Hong Kong-Macao Greater Bay Area Urban Agglomeration” should be set up to supervise the implementation of synergetic policies and measures, and restrict the behaviors of all partners according to the agreement. In addition, we should also construct a multi-level interest compensation mechanism in Greater Bay Area, coordinate the overall interests and local interests of the urban agglomeration in Greater Bay Area, and improve the motivation and willingness of the synergetic development of the three places.
2. Constructing an integrated market in Greater Bay Area and accelerating the connection of business environment and rules: it is suggested that the duty-free policy for products originating in Hong Kong and Macao under the CEPA agreement should be extended to all products from Hong Kong and Macao, including imports from Hong Kong and Macao. We should continue to deepen the implementation of the measures for the opening of service trade under the CEPA agreement, try to cancel the restrictions on the share ratio and access conditions for the investors of Hong Kong and Macao to enter the finance, telecommunications, culture, and other fields of the Guangdong Pilot Free Trade Zone, relax the approval of investment fields and qualifications, cancel the foreign exchange management system, and construct an integrated financial market among the three regions. We should also encourage the telecommunication companies in the three places to cooperate in a win-win way and construct an interconnected, convenient and smooth Internet and communication network in Greater Bay Area, promote the electronization and automation of endorsement and customs clearance in the three places, speed up the construction of self-service customs clearance and inspection channels, and realize “one endorsement, multiple trips.” In addition, it is necessary to accelerate the formulation of unified industry service standards, professional qualifications and professional titles in Greater Bay Area, carry out pilot work of mutual recognition of professional qualifications in Greater Bay Area, explore the establishment of a list of professional qualifications of Guangdong, Hong Kong and Macao talents, and study and carry out mutual recognition of academic qualifications in the three places. We need to increase efforts to replicate and promote the successful experience of the reform in the areas of commercial registration, in-process and after event supervision in the free trade zone to other cities in the Greater Bay Area, and gradually improve the legal, international, and convenient business environment. Finally, we should reform the customs management system and the setting of layout, speed up the realization of the goal of “Liberalizing the First Line and Managing the Second Line” in Greater Bay Area, and promote the innovative models such as “one place, two inspections” and “cooperative inspection” [16].
3. Constructing a reasonable division of labor system and form a regional community of interests: based on the existing two-wheel drive model of the advanced manufacturing and the modern service industry, a division of labor system of Greater Bay Area urban agglomeration with complementary advantages and misplaced competition will be formed to comprehensively improve the overall competitiveness. Guangzhou should play the central role as the “headquarters,” maintain its radiation and diffusion capacity to the Pearl River Delta, and promote the upgrading of industrial structure by cultivating strategic emerging industries such as biomedicine, new materials, new energy, and marine biology. Shenzhen can make use of its high-tech industrial characteristics to improve the scientific and technological content and innovation level of manufacturing industry, realize the strong combination of technological innovation and financial industry, and form complementary advantages with Hong Kong. Foshan, Dongguan, and Zhongshan can accelerate the landing and transformation of high-tech achievements from Guangzhou and Shenzhen, and accelerate the transformation and upgrading of traditional manufacturing industries. Huizhou, Jiangmen, and Zhaoqing should actively undertake the transfer of relevant industries to achieve dislocation development. Hong Kong and Macao should actively integrate into the overall development situation of the Greater Bay Area, and take advantage of their service industry advantages to drive the transformation and upgrading of the manufacturing industry and the development of modern service industry in the Pearl River Delta. At the same time, we should consolidate and enhance the status and functions of Hong Kong as an international financial center, shipping center, and trade center, continue to give full play to the unique advantages of Hong Kong and Macao as “super contacts,” and build the Great Bay Area into a high-level opening-up platform[17].
4. Constructing a synergetic innovation system and creating a first-class science and technology bay area: innovation is the driving force for the synergetic development of the regional economy. We should strengthen the integration of innovation resources in the three regions, optimize the innovation environment, and construct a synergetic innovation system for Greater Bay Area. The major universities and scientific research institutes in Guangzhou can strengthen the research and exploration of basic frontier fields and the education and training of innovative talents. Tianhe High Tech Development Zone, science city, and other advantageous strategic platforms should strengthen the joint innovation with advanced manufacturing industries in Foshan, Dongguan, and Zhongshan. Shenzhen should focus on the development of high-tech industries and strive to achieve breakthroughs of the core technologies in the fields of new energy, new technology, biomedicine, energy conservation, and environmental protection. Many well-known universities and scientific research institutions in Hong Kong should strengthen their cooperation with universities and enterprises in the Pearl River Delta to accelerate the implementation and transformation of scientific research achievements while improving research and development capabilities. The three places should jointly construct a technological innovation service platform for Greater Bay Area, especially for small and medium-sized enterprises, to promote the smooth implementation of enterprise innovation activities and technological upgrading. The financial institutions should be encouraged to introduce capital into the process of industrial incubation and transformation of innovative technologies, and provide targeted financial products and services. The connection of intellectual property protection mechanisms among the Pearl River Delta, Hong Kong, and Macao should be accelerated to optimize the external environment for scientific and technological innovation. Finally, we should take advantage of preferential policies in registered residence, taxation, customs clearance, and other aspects to attract innovative talents and innovative enterprises to gather and flow freely in Greater Bay Area.

## Figures and Tables

**Table 1:** Evaluation Indicator System of Regional Economic synergetic Development.

System	Subsystem	Target layer	Indicator layer	Unit	Attribute
Economic synergetic development system of Guangdong-Hong Kong-Macao greater bay area	Economic scale subsystem	Economic aggregate	GDP sum	RMB 100 million	Positive
		Government revenue	Fiscal revenue	RMB 100 million	Positive
		Social consumption	Total retail sales of consumer goods	RMB 100 million	Positive
		Capital investment	Investment in fixed assets	RMB 100 million	Positive
		Labor input	Total employment	10,000 people	Positive
	Economic quality subsystem	Growth potential	Per capita GDP	RMB	Positive
		Industrial structure	Proportion of tertiary industry in GDP		Positive
		Production efficiency	Labor productivity		Positive
		Level of consumption	Final consumption rate		Positive
		Employment level	Unemployment rate		Positive
	Sustainable development subsystem	Input for scientific research	Proportion of scientific research in GDP		Positive
		Educational input	Proportion of education expenditure in GDP		Positive
		Environmental protection input	Proportion of environmental protection expenditure in GDP		Positive
		Medical input	Number of sickbeds per 10,000 people	Beds per 10,000 people	Positive
	Element flow subsystem	Personnel turnover	Average daily passenger flow	10,000 person times per day	Positive
		Goods flow	Average daily freight volume	10,000 tons per day	Positive
		Capital flow	Per capita FDI stock	Yuan per person	Positive
		Information flow	Number of mobile phones per 100 people	Households per 100 people	Positive
	Market environment subsystem	Opening up of foreign trade	Ratio of dependence on foreign trade		Positive
		Opening up of foreign capital	Opening ratio of foreign capital		Positive
		Opening up of tourism	Opening ratio of tourism		Positive
		Government size	Proportion of government consumption expenditure in GDP		Negative
		Financial environment	Per capita loan scale	Yuan per person	Positive

**Table 2:** The Order Degree of Subsystems of Economic synergetic Development of Guangdong-Hong Kong-Macao Greater Bay Area Urban Agglomeration from 2007 to 2019.

年份	*μ*(*b*_1_)	*μ*(*b*_2_)	*μ*(*b*_3_)	*μ*(*b*_4_)	*μ*(*b*_5_)
2007	0.4818	0.4262	0.2737	0.4000	0.3592
2008	0.5323	0.5999	0.2811	0.3838	0.3599
2009	0.5984	0.5065	0.3612	0.3826	0.3591
2010	0.6295	0.4992	0.3573	0.3862	0.3855
2011	0.6417	0.4442	0.4074	0.3565	0.3986
2012	0.6518	0.4876	0.4694	0.4913	0.4030
2013	0.7013	0.5095	0.5102	0.4579	0.4019
2014	0.6915	0.5508	0.4562	0.4646	0.4292
2015	0.7144	0.5605	0.4280	0.4695	0.4318
2016	0.7164	0.5611	0.3807	0.4459	0.4432
2017	0.7533	0.5732	0.4227	0.5315	0.4695
2018	0.7962	0.6584	0.4198	0.5963	0.5173
2019	0.8157	0.6794	0.4161	0.6395	0.5350

**Table 3:** Temporal and Spatial Changes in the Level of Synergistic Economic Development of Guangdong-Hong Kong-Macao Greater Bay Area Urban Agglomeration.

Year	E _Guangdong- Hong Kong-Macao Greater Bay Area Urban Agglomeration_	E _core cities_	E _sub-core cities_	E _non-core cities_
2008	−0.0123	0.0838	0.2555	−0.0774
2009	−0.0132	0.1626	0.2893	−0.0856
2010	0.0413	0.1442	0.3387	0.0389
2011	0.0786	0.1737	0.3236	0.0216
2012	−0.0875	0.1023	0.2589	−0.0943
2013	−0.0734	0.1298	0.2799	−0.2040
2014	0.0843	0.1573	0.3823	0.0693
2015	0.0909	0.1992	0.3785	0.0775
2016	0.0805	0.1828	0.3860	0.0796
2017	0.1225	0.2197	0.3854	0.0413
2018	0.1799	0.2440	0.3966	0.1088
2019	0.2160	0.2534	0.4024	0.1143

**Table 4:** The level and division standard of regional economic synergetic development.

Level	Economic synergy level	Division standard
1	−1.0–0	Unsynergetic
2	0–0.1	Low degree of synergy
3	0.1–0.2	Reluctant synergy
4	0.2–0.4	Primary synergy
5	0.4–0.6	Intermediate synergy
6	0.6–0.8	Good synergy
7	0.8–1.0	Complete synergy

**Table 5:** Synergy degree of composite system of economic synergetic development of three major urban agglomerations from 2008 to 2019.

Year	*E *_Guangdong-Hong Kong-Macao Greater Bay Area_	*E *_Yangtze River Delta urban agglomeration_	*E *_Beijing-Tianjin -Hebei urban agglomeration_
2008	−0.0123	0.2493	0.1167
2009	−0.0132	0.3138	0.1692
2010	0.0413	0.2306	0.2259
2011	0.0786	0.2721	0.2542
2012	−0.0875	0.3104	0.3007
2013	−0.0734	0.3539	0.2730
2014	0.0843	0.4375	0.2593
2015	0.0909	0.4267	0.3428
2016	0.0805	0.4190	0.3297
2017	0.1225	0.4572	0.4180
2018	0.1799	0.5227	0.3755
2019	0.2160	0.5194	0.4003

## Data Availability

The data set can be accessed upon request.
